# Physiological comfort evaluation under different airflow directions in a heating environment

**DOI:** 10.1186/s40101-022-00289-x

**Published:** 2022-04-15

**Authors:** Kaori Tamura, Sayaka Matsumoto, Yu Hsuan Tseng, Takayuki Kobayashi, Jun’ichi Miwa, Ken’ichi Miyazawa, Soichiro Matsumoto, Seiji Hiramatsu, Hiroyuki Otake, Tsuyoshi Okamoto

**Affiliations:** 1 Faculty of Arts and Science, Kyushu University, 744 Motooka, Nishi-ku, Fukuoka, 819-0395 Japan; 2 Department of Information and Systems Engineering, Faculty of Information Engineering, Fukuoka Institute of Technology, 3-30-1 Wajiro-higashi, Higashi-ku, Fukuoka, 811-0295 Japan; 3 Graduate School of Systems Life Sciences, Kyushu University, 744 Motooka, Nishi-ku, Fukuoka, 819-0395 Japan; 4 Mitsubishi Heavy Industries Thermal Systems LTD., 3-1, Asahi, Nishi-biwajima-cho, Kiyosu, Aichi 452-8561 Japan; 5 Research and Innovation Center, Mitsubishi Heavy Industries Ltd., 1, Aza Kanda, Iwatsuka-cho, Nakamura-ku, Nagoya, Aichi 453-8515 Japan

**Keywords:** Comfort evaluation, Airflow direction, Electroencephalogram, Respiration, Heating environment

## Abstract

**Background:**

Indoor airflow and thermal comfort are difficult to assess through subjective evaluations because airflow sensations can differ based on various factors, such as personal characteristics, interests, preferences, and the current state of mind. Thus, subjective evaluations should be combined with objective assessments, such as physiological measurements. This study evaluated airflow and thermal comfort through physiological measurements, including skin temperature, electroencephalography, respiration, and electrocardiography, in addition to subjective evaluations.

**Methods:**

Twenty participants entered a test room at 30 °C after staying in an acclimation room at 18 °C for 20 min. They were exposed to *indirect* and *direct* airflow toward their faces and performed four tasks under each condition: resting, counting to 10 s following time alerts, counting to 10 s in the mind, and mental calculation. The mean speed of the air directed to the participants’ faces was 0.123 m/s and 0.225 m/s in the *indirect* and *direct* conditions, respectively.

**Results:**

The gamma and beta bands of electroencephalograms taken at the left-temporal (T3) and left-parietal (P7) sites showed significantly lower amplitudes under the indirect condition (gamma, T3: *p* = 0.034, P7: *p* = 0.030; beta, T3: *p* = 0.051, P7: *p* = 0.028). Similarly, the variability of respiration was lower under the indirect condition (*p* < 0.010). The amplitudes of gamma and beta waves showed significant correlations with anxiousness levels (gamma, T3: *r* = 0.41; beta, T3: *r* = 0.35).

**Conclusions:**

Our results suggest that indirect heating airflow causes lower mental stress and fatigue than those induced by direct flow, which is equivalent to more comfort. The results of this study suggest that physiological measurements can be used for the evaluation of unconscious indoor comfort, which cannot be detected by subjective evaluations alone.

**Supplementary Information:**

The online version contains supplementary material available at 10.1186/s40101-022-00289-x.

## Background

Airflow movement and direction can influence the comfort level in a heating environment [1–4]. When evaluating thermal comfort, air movements are regarded as unwanted because high wind velocities decrease indoor comfort [1, 3]. Satisfaction with airflow direction will improve quality of life and promote better productivity in offices and schools.

Discomfort under different indoor airflow velocities has long been studied, and satisfaction varies depending on temperature, climate, and individuals [5]. Some studies have investigated the preferences for airflow movement through subjective votes, showing that air movement was preferred when people felt warm or slightly warm [6–8]. On the other hand, a study in subtropical regions showed that the preferences for air movement largely differed between the responders [9], and another report from a subtropical region showed that fewer people preferred air movement when the room temperature in an air-conditioned environment increased [10]. Other studies have also investigated the effects of the velocity and direction of the heating airflow. They showed that more people favored a lower warm airflow velocity [11] and that warm airflow directed to the face induced uncomfortable feelings [12]. Subjective evaluations can vary intra- and inter-individually according to physical and mental states. Thus, objective measurements should be added to the conventional subjective approach for a more robust evaluation of thermal comfort.

There are many subjective but few objective approaches for evaluating feelings of comfort depending on airflow conditions. This imbalance is one possible reason that there is no consensus on how airflow affects comfort. For example, Yamashita et al. found a non-linear relationship between airflow temperature and subjective comfort, while they found a linear relationship between airflow temperature and thermal sensation [13]. Cui et al. evaluated the effects of heating airflow on cognitive tasks, but there was no significant difference in the performance levels [14]. These reports highlight the difficulty in evaluating the effects of airflow on mental states through subjective assessments and behavioral measurements.

Conventionally, to assess indoor pleasantness, including airflow comfort, subjective assessments or rating scales are widely used. A common international scale called predicted mean vote (PMV) [15] was established to summarize and standardize such assessments. However, the validity of PMV is controversial [16, 17], especially in East Asian regions [18]. The reported predicted accuracy of the actual votes involving several building types was 34% [17]. Van Hoff summarized the discrepancies between PMV prediction and actual votes of the participants, showing that the preferences for the indoor environmental conditions varied individually and were dissociated from those based on the PMV model in Asian studies [16]. Differences between PMV and actual mean vote in cases of naturally ventilated and air-conditioned spaces have been reported as well. Humphreys and Nicol reported that there were underlying biases concerning all variables contributing to the PMV calculation. For example, when the airflow speed exceeded 0.2 m/s, the divergence between PMV and actual votes tended to be wider [19]. PMV is one of the typical scales that aim at estimating a mean subjective comfort evaluation, which may provide a misleading prediction of the comfort level when considering the effect of airflow direction. Objective parameters such as physiological measurements should be added to the conventional subjective evaluation.

Electroencephalography (EEG) is a strong candidate for the objective evaluation of airflow comfort. Some components of EEG signals are known to directly reflect the mental state [20, 21]. For example, a previous study found that the beta and gamma wave amplitudes recorded from the participants were higher when they were subjected to direct airflow than when airflow was not provided [22], thus demonstrating that these measurements could be useful indicators to evaluate comfort feelings with different airflow conditions. Such results were also confirmed in a more recent study that investigated airflow comfort in a cooling environment using EEG measurements [23], and this same methodology is used in this study to estimate the differential effects of either direct or indirect heating airflows on comfort feelings [23]. Some conventional studies have shown that direct and fast warm airflow induced uncomfortable feelings [11, 12], but others reported that air movement was preferred [6–8]. Heating airflow comfort needs to be evaluated through various methods, such as physiological measurements and subjective assessments, to confirm whether similar EEG patterns can be observed under cooling and heating airflows. In this study, we hypothesized that indirect heating airflow will induce lower mental stress than direct airflow, which will be reflected in lower beta and gamma EEG amplitudes. According to our studies [22, 23], the beta and gamma amplitudes can be useful to assess airflow comfort under a heating environment as well.

The activity of the central nervous system can be reflected in EEG signals and that of the autonomic nervous system can be related to airflow comfort. In addition to EEG, other physiological measurements, such as respiration and electrocardiography (ECG), are useful to estimate comfort states. Respiration has previously been measured to evaluate the physiological effects of indoor environments [24, 25]. It has been suggested that respiration variability increases due to spontaneous sighs under mental stress tasks [26, 27] and that it reflects cognitive states, including sustained attention, cognitive loads, and mental stress [26–28]. By contrast, ECG parameters can indicate the activity of the autonomic nervous system. The heart rate variability calculated from ECG signals is indicative of parasympathetic and sympathetic activity [29]. The combination of physiological measurements can enable the evaluation of airflow comfort based on both the central and autonomic nervous systems.

The main purpose of this study was to evaluate how the direction of airflow produced by air conditioners influences the feeling of comfort in a heating environment through subjective and objective measurements, including EEG, respiration, ECG, and other behavioral and physiological responses. These measurements were performed with heated air flowing either directly or indirectly into the participants’ faces. The physiological measurements were used alongside subjective assessments to evaluate the comfort as a function of airflow direction.

## Materials and methods

### Participants

We recruited 20 university students to participate in our experiments (female, *n* = 8; male, *n* = 12). Their mean age, mean body weight, and mean height were 21 ± 2.1 years, 57.9 ± 8.1 kg, and 167.6 ± 8.1 cm, respectively. None of the participants self-reported having smoking habits or neurological deficits. Four female participants self-reported that their basal body temperatures were lower due to the follicular phase during the experiment.

The sample size was determined according to the standard of neurophysiological studies: about 20 participants, though behavioral or questionnaire studies, can provide a larger sample size of at least 150–250 data points. The typical sample size of neuroscience studies involving EEG measurements tends to be low because of the need for considerable time, investment, and resources per participant, as well as more complex measurement tools than required for behavioral studies [30, 31]. Moreover, ethical issues should be considered in neurophysiological studies. Bacchetti et al. suggested that the projected burden per participant would be more suitable for studies with a smaller sample size [32]. Thus, we recruited 20 participants for this study according to the feasibility and ethical issues of neurophysiological studies.

The experiments were approved by the joint ethics committee of the Faculty of Arts and Science and the Center for Health Sciences and Counseling from Kyushu University (201815, 201815-1). Written informed consent was obtained from each participant. All methods were performed per the approved guidelines.

### Apparatus and protocol

The experiments were conducted in an experiment room at Mitsubishi Heavy Industries LTD., Aichi, Japan. Due to a schedule conflict between participants and the experiment room, the tests were performed in two periods, from 21 January 2019 to 1 February 2019 and from 10 May 2019 to 23 May 2019. The data collected in the two periods were pooled. The environmental parameters, including room temperature, wind direction, and wind velocity, were monitored and controlled simultaneously from a control room outside the experiment area.

**Fig. 1:**
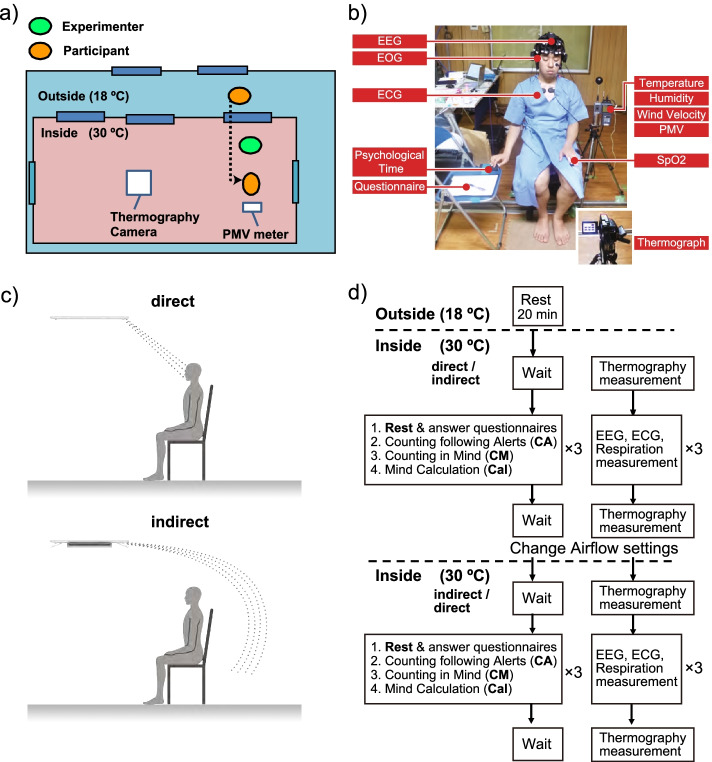
Experimental environment, settings, and procedure. **a** Experimental environment. **b** Location of the measuring sensors and PMV meter. A thermography camera was located in front of the participants. **c** Airflow settings for the two surveyed conditions: direct and indirect. **d** Experimental procedure. The left side indicates the task sequence in an experiment, and the right side indicates the sequence of physiological measurements corresponding to the left tasks. The task and measurement sequences were performed at the same time. The order of airflow settings was pseudo-randomized across participants. We obtained permission from the participants for printing the images of their faces. This figure was modified from Tamura et al. [23] licensed under CC BY 4.0 (https://creativecommons.org/licenses/by/4.0/)

The indoor climate condition was controlled using a commercially available air conditioner (Draft Prevention Panel, Mitsubishi Heavy Industries Thermal Systems LTD., Tokyo, Japan) with special panels to avoid direct impact of air from the indoor unit. Two different airflow conditions were set as follows (see Fig. 1): (1) direct airflow, where the current from the air conditioner was set to hit the participants’ faces, and (2) indirect airflow, in which the air was diverted by the control panels (Fig. 1c). The staff stayed in the control room to monitor the physical parameters measured indoors and to control the air conditioning unit.

To simulate the outdoor temperature during winter in Japan, each participant was asked to stay for 20 min in a waiting room with the temperature set to 18 °C. This acclimation to a cool temperature counteracted the effect of variations in outside temperature during the experiment period. After the acclimation, each participant moved to the experiment room, with the room temperature set to 30 °C. The temperature in the experiment room simulated a heating environment during winter in Japan. EEG and ECG electrodes were attached to each participant within approximately 10 min after entering the experiment room. The participants then performed tasks under the different airflow conditions (Fig. 1d). The order of airflow conditions was pseudo-randomized across the participants. The air conditioner was controlled before taking measurements under each condition.

The participants performed the same sequence of tasks used by the authors in a previous study [23]. The sequence consisted of four assignments: rest for 1 min, time counting following alerts (CA) for 1 min, time counting in mind (CM) for 1 min, and mental calculation (Cal) for 1 min. Each task was performed after a short instruction whose duration was about 40 s. This sequence was repeated three times in a random order for both the direct and indirect airflow conditions (Fig. 1d). Thus, the total duration per airflow condition was (1 min + 40 s) × 4 tasks × 3 times = 20 min. During the rest period, the participants were instructed to keep their eyes closed for 1 min. After resting, each participant answered a questionnaire (see the “Subjective assessments” section).

The CA and CM assignments were designed to measure psychological time. In the CA task, each participant was required to press a button following time alerts every 10 s for 60 s. In the CM task, each participant was asked to estimate 10 s as exactly as possible, press the button after counting for 10 s in their mind (without uttering words), and repeat for six times (~ 60 s).

In the Cal assignment, each participant was asked to consecutively subtract 13 from a four-digit number (e.g., 1012 − 13 = 999, 999 – 13 =…) as quickly as possible for 60 s. The four-digit number was randomly assigned, ranging from 1000 to 1012.

Each participant sat in a chair placed at the designated position near an experimenter. An anemometer was placed close to each participant, and a thermography camera was placed in front of them (Fig. 1a, b; see details in the “Physical measurement and analysis” and “Wind velocity measurement” sections). The participants were asked to wear the same type of hospital gown to standardize the clothing. The peripheral oxygen saturation (SpO_2_) was measured with a finger pulse oximeter to confirm safe oxygen levels of more than 95% (Fig. 1b).

### Physical measurement and analysis

During the experiments, room temperature, relative humidity, and wind velocities were measured with a PMV meter (AM-101, Kyoto Electronics Manufacturing Co. Ltd., Japan) placed close to the participant. To analyze the changes in time series data, each parameter was segmented and averaged across the three repetitions of each airflow condition. One experiment failed to record PMV data in the direct airflow condition; therefore, the number of PMV data under the direct setting was 19, while the remaining physical data were recorded 20 times in each condition. To compare the mean parameters, paired *t*-tests were performed on the averaged data. The statistical analyses were conducted using JMP® 14SW (SAS Institute Inc., Cary, NC, USA).

### Wind velocity measurement

**Fig. 2:**
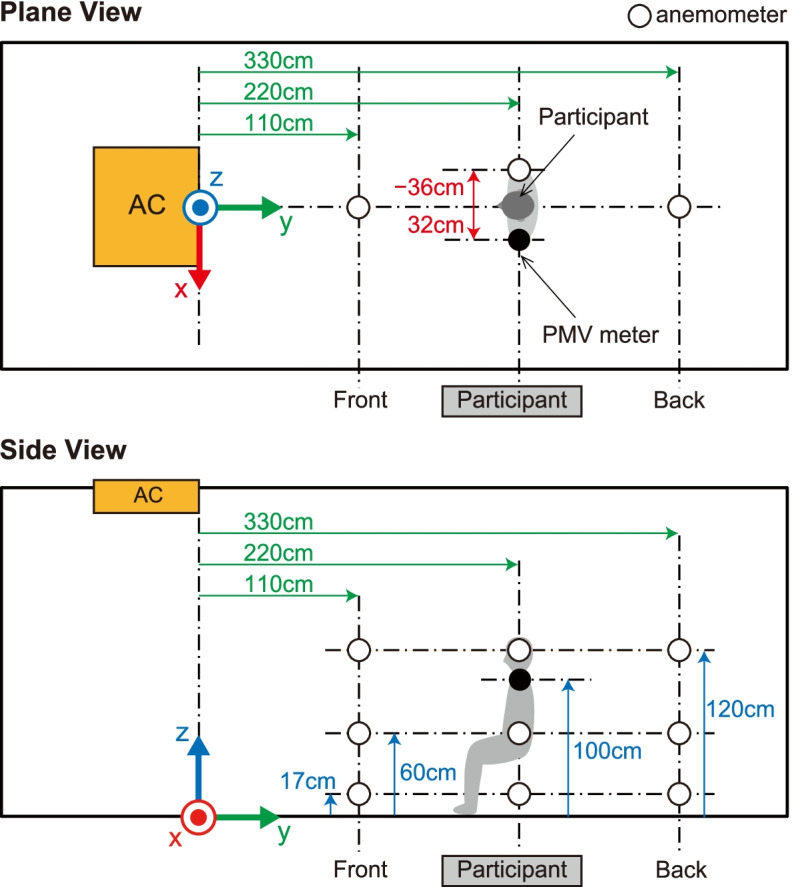
Measurement settings of wind velocity and direction. White circles indicate the nine locations where the wind velocity and direction were measured using an anemometer. The nine locations were combinations of three positions in the front-back direction and three heights. The black circle indicates the location of the PMV meter. AC indicates the location of the air conditioner. This figure was reprinted from Tamura et al. [23] licensed under CC BY 4.0 (https://creativecommons.org/licenses/by/4.0/)

To ensure the correct environment for physiological measurements of the participants, wind velocity and direction were measured using a multi-channel anemometer (Multi-channel anemometer 1550 series, KANOMAX JAPAN INC., Osaka, Japan) and probe cables (MODEL 1504 probe cable velocity channel, KANOMAX JAPAN INC., Osaka, Japan). The anemometer was set up at nine points (Fig. 2). The minimum distance between the floor and the anemometer was 17 cm due to the anemometer’s size. The measurement was controlled by an accessory software (MODEL S620-00 Ver 1.46, KANOMAX JAPAN INC., Osaka, Japan). The wind velocity and direction were measured for 1 min at a sampling rate of 10 Hz after waiting a few minutes for the machine to stabilize. The sampled wind velocity data in each direction were averaged for the 1-min measurements.

### Thermography measurement and analysis

To assess the skin temperature, thermography data were measured before and after each airflow setting (InfReC R550, NIPPON AVIONICS Co. LTD., Tokyo, Japan). In each thermographic image, the face area (178 × 140 pixels) was determined and cropped. The thermal data in this area were averaged, and a two-way within-subject analysis of variance (ANOVA) was conducted (airflow conditions × before/after measurement).

### EEG and ECG

The EEG and ECG data were measured during the four tasks under both airflow conditions. The measured data were transformed using a discrete Fourier transform (DFT) to extract EEG amplitudes or heart rate variability. The extracted parameters were compared between the airflow directions using ANOVA. The detailed methods of measurement, preprocessing, and statistical analysis are described as follows.

We recorded EEG data across 19 channels, as per the international 10–20 system [33], using dry active electrodes, CGX Flex, and Drypad sensors with a wireless headset (Quick-20, CGX, San Diego, USA). The reference electrode was placed on A1, as defined by the above system, and an additional electrode was placed on A2 for further analysis. To remove artifacts, electrooculogram (EOG) data were also collected using AIM Generation 2 (CGX, San Diego, USA) by adding sensors to the headset. Bipolar horizontal and vertical EOG data were recorded by two additional electrodes placed below the left eye and laterally from the outer canthus of the right eye, with a reference electrode placed on the forehead. The EEG and EOG data were amplified and digitalized at a sampling rate of 500 Hz.

In addition to EEG and EOG channels, we recorded ECG data using AIM Generation 2 (CGX, San Diego, USA). ECG data were measured by the bipolar limb lead method, which used a positive electrode on the left arm and a negative electrode on the right one with a reference electrode. These data were sampled at 500 Hz.

The measured EEG data were re-referenced to the averages of A1 and A2 and separated by task to reduce EOG artifacts efficiently. The EEG data during each session of 60 s were segmented and filtered with a notch filter of 60 Hz. The data were then transformed using a DFT with a rectangular window to obtain the DFT coefficients. DFT analysis was performed using the “fft.m” function in MATLAB (MathWorks, Inc., Natick, USA). Amplitudes in the frequency bin calculated from the DFT coefficients were averaged within the beta (14 ≤ *f* < 30 Hz) and gamma (30 ≤ *f* < 50 Hz) frequency bands, where *f* represents the frequency. The segmented data were rejected when the EOG or EEG data exceeded ± 100 μV to eliminate eye blinking artifacts. The mean rates of segments that remained after rejection were 77.1 ± 11.7% and 80.0 ± 10.0% in direct and indirect airflow conditions, respectively. Changes in each oscillation band of the EEG time series were assessed using a two-way ANOVA (airflow conditions × four tasks) for each electrode. We did not conduct any post hoc testing because our purpose was to determine the main effect of airflow conditions on EEG.

From the EEG data, gamma and beta frequency bands were analyzed according to our previous studies [22, 23], which examined the relationship between indoor airflow comfort and the gamma and beta bands. The results indicated that the amplitudes of these waves decreased as airflow comfort improved [23]. In this study, the analytic EEG electrodes were determined by a data-driven method, as it was not possible to select an analytic region based on previous research, which has found various regions to be the areas related to thermal comfort. For example, an analysis of functional magnetic resonance imaging (fMRI) reported that thermal sensation and thermal comfort were processed by different brain networks [34]. Thus, the 19 electrodes herein used were screened by subtracting the amplitudes measured under the direct condition from those under the indirect condition. Based on this criterion, electrodes T3 and P7 were identified as the analytic electrodes for the betta and gamma waves.

ECG data corresponding to each 60-s session were segmented, detrended, and smoothed by a moving window. R-R intervals were calculated for all data by finding R peaks. Then, a DFT was applied to the R-R interval data to obtain heart rate variability. Using the DFT components from the R-R interval data, we calculated the mean components for high frequency (HF) (0.15 ≤ *f* < 0.4 Hz) and low frequency (LF) (0.04 ≤ *f* < 0.15 Hz). LF/HF was used as an indicator of sympathetic modulation, and HF as an indicator of parasympathetic activity. We conducted a two-way ANOVA to assess HF and LF/HF data across the two airflow conditions and the four tasks.

### Respiratory waveform

We recorded respiration waveforms using AIM Generation 2 and spatial bio-impedance-based respiration sensors (CGX, San Diego, USA) during the four tasks. The two respiration sensors were placed on the left and right sides of the participant’s chest. These data were sampled at 500 Hz.

The respiration waveforms during each 60-s session were segmented. Then, a DFT was applied to obtain the peak frequency of the power spectral density. From the power spectral density, the full width at half maximum (FWHM) of the peak frequency was calculated. A smaller FWHM indicates a more regular breathing state at the peak frequency of the respiration wave, while a larger FWHM indicates more irregular breathing. We used FWHM as a parameter to estimate the relaxation state, with a low mental load corresponding to regular breathing. A two-way within-subject ANOVA was then conducted (airflow conditions × four tasks).

### Subjective assessments

Between resting and CA, each participant answered a questionnaire rating thermal sensation (1, very cold; 7, very hot), pleasantness (0, very unpleasant, 100, very pleasant), fatigue (0, best condition and no fatigue; 100, worst condition and extreme fatigue), sleepiness (1, fully awake; 9, very sleepy), and anxiousness (0, not feeling anxious at all; 100, feeling extremely anxious). The thermal sensation and sleepiness were assessed with 7- and 9-step Likert scales, respectively, and the remaining factors were assessed with horizontal visual analog scales (VAS) with 10-cm lines (Table 1).

**Table 1:** Questionnaire structure

Category	Scale	Type
Thermal sensation	Very cold ←→ very hot (1–7)	7-step Likert, integer values
Pleasantness	Very unpleasant ←→ very pleasant (0–100)	10-cm VAS
Fatigue	Best condition and no fatigue ←→ worst condition and extreme fatigue (0–100)	10-cm VAS
Sleepiness	fully awake ←→ very sleepy (1–9)	9-step Likert, integer values
Anxiousness	Not feeling anxious at all ←→ feeling extremely anxious (0–100)	10-cm VAS

The responses to the questionnaire were taken after resting and were analyzed using a *t*-test. The subjective assessments were obtained by computing the correlations with the analytic EEG gamma and beta amplitudes at T3 and P7, using pairwise Pearson’s coefficients within participants. The EEG amplitudes were averaged across the four tasks.

### Psychological time

Psychological time has been discussed in relation to negative emotions, including boredom [35, 36], which will lead to overestimating the passage of time [37]. In this study, we measured psychological time as an indicator of psychological stress caused by airflows. The analytic psychological time was defined as the mean response duration in each CA and CM task. A positive psychological time indicates that the individual perceived a slow pace of time. A two-way ANOVA was performed across the two airflow conditions and the two assignments (CA and CM).

### Correlation between the EEG and subjective assessments

The correlations between EEG amplitudes and subjective assessments were performed by pairwise correlation. The EEG amplitudes were averaged across the four tasks and sessions in each channel, frequency band, and airflow condition. Then, the EEG and subjective assessment results were used for pairwise correlation analysis within participants to obtain correlation coefficients and *p*-values, using the “corrcoeff.m” function in MATLAB (MathWorks, Inc., Natick, USA).

## Results

### Wind velocity and direction

**Fig. 3:**
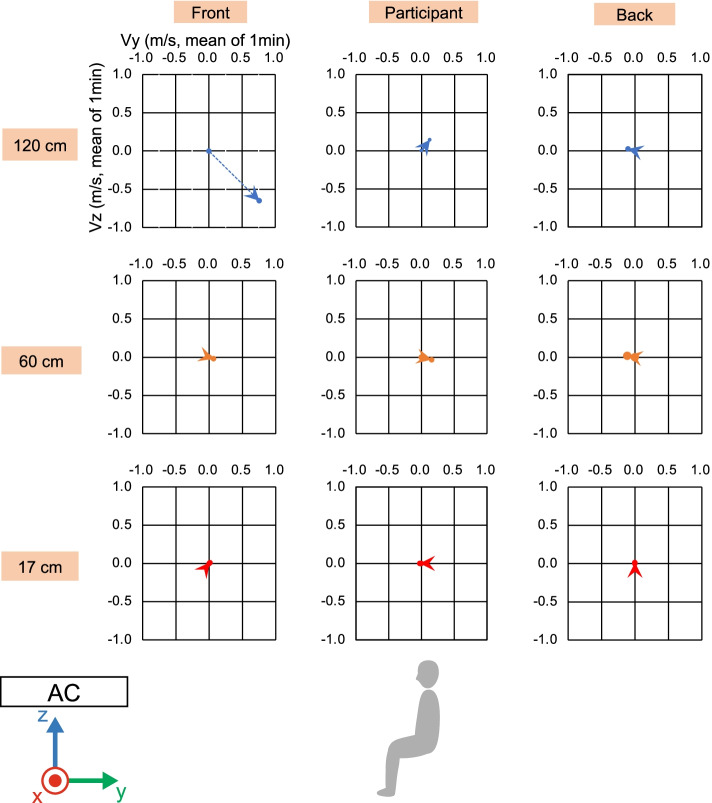
Wind velocity and direction in the *Y*-*Z* plane under direct airflow. *Vy* and *Vz* indicate the mean of the 1-min velocity in the *Y* and *Z* directions, respectively. Dotted lines indicate the vectors of the wind velocities

**Fig. 4:**
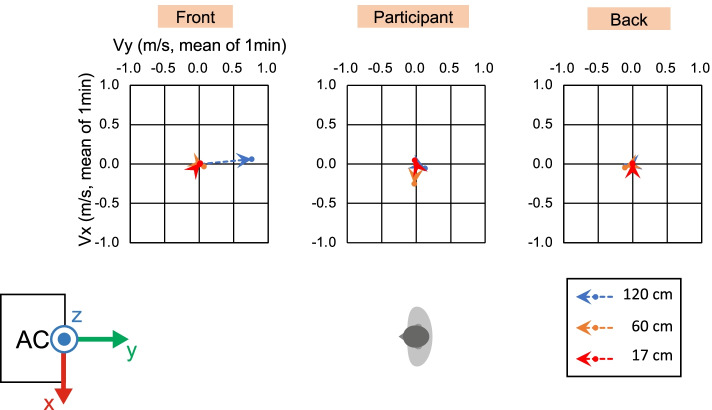
Wind velocity and direction in the *X*-*Y* plane under direct airflow. *Vx* and *Vy* indicate the mean of the 1-min velocity in the *X* and *Y* directions, respectively. Dotted lines indicate the vectors of the wind velocities

**Fig. 5:**
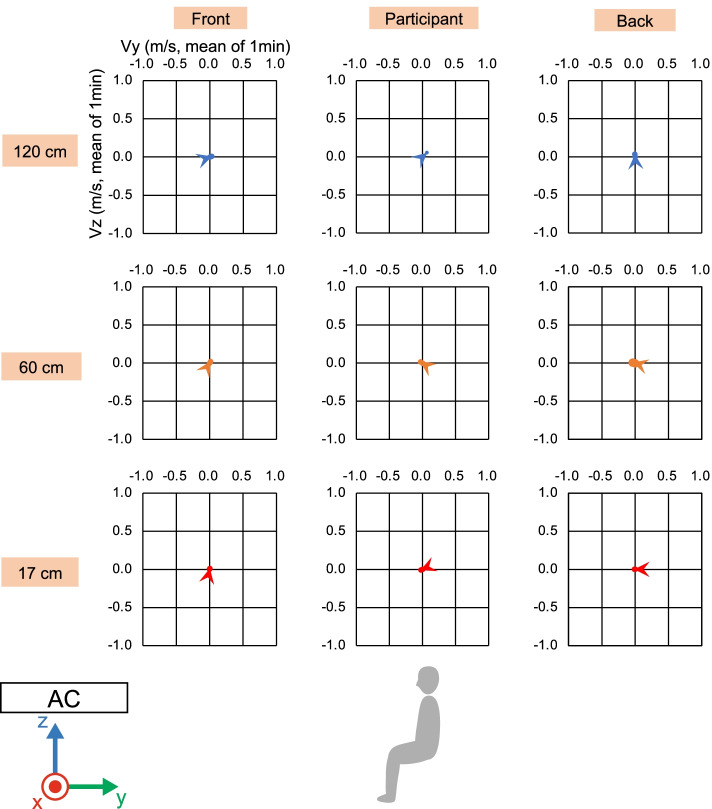
Wind velocity and direction in the *Y*-*Z* plane under indirect airflow. *Vy* and *Vz* indicate the mean of the 1-min velocity in the *Y* and *Z* directions, respectively. Dotted lines indicate the vectors of the wind velocities

**Fig. 6:**
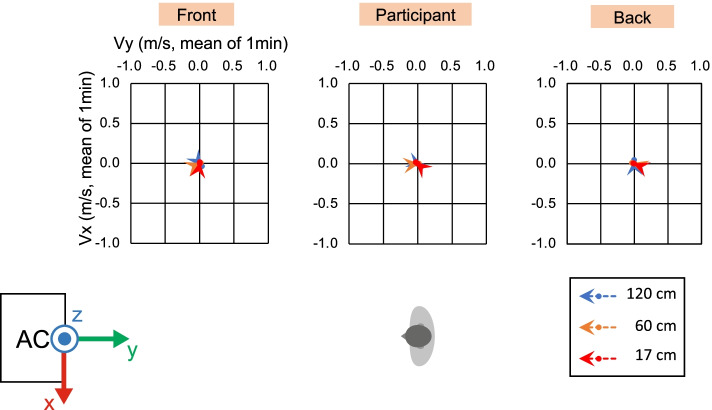
Wind velocity and direction in the *X*-*Y* plane under indirect airflow. *Vx* and *Vy* indicate the mean of the 1-min velocity in the *X* and *Y* directions, respectively. Dotted lines indicate the vectors of the wind velocities

To ensure the correct experimental environment, we measured the wind velocity and direction under direct and indirect airflow conditions (Figs. 2, 3, 4, 5, and 6). In front of the participant, at a height of 120 cm from the floor, the wind velocity under direct current was larger than that under indirect airflow, especially in the *Y*- and *Z*-axis directions (Tables 2 and 3, Figs. 3 and 5: 120 cm at the front). These data indicate that the wind direction and velocity differed between the two conditions, especially around the sitting sites of the participants.

**Table 2:** Wind velocity under direct airflow

Height [cm]	Location	Wind velocity [m/s]/mean of 1 min: *X*	Wind velocity [m/s]/mean of 1 min: *Y*	Wind velocity [m/s]/mean of 1 min: *Z*	Wind velocity [m/s]/mean of 1 min: composite
120	Front	0.063	0.761	− 0.651	1.017
120	Participant	− 0.055	0.125	0.143	0.225
120	Back	− 0.024	− 0.103	0.025	0.124
60	Front	− 0.031	0.068	− 0.019	0.177
60	Participant	− 0.248	0.155	− 0.030	0.340
60	Back	− 0.043	− 0.113	0.019	0.131
17	Front	0.011	0.011	0.012	0.038
17	Participant	0.050	− 0.023	0.000	0.073
17	Back	0.014	0.000	0.012	0.029

The mean of 1-min wind velocity [m/s] in each direction and composite vector length under the direct condition at each measurement position

**Table 3:** Wind velocity under indirect airflow

Height [cm]	Location	Wind velocity [m/s]/mean of 1 min: *X*	Wind velocity [m/s]/mean of 1 min: *Y*	Wind velocity [m/s]/mean of 1 min: *Z*	Wind velocity [m/s]/mean of 1 min: composite
120	Front	− 0.035	0.031	0.009	0.121
120	Participant	− 0.024	0.068	0.051	0.123
120	Back	0.050	− 0.002	0.034	0.076
60	Front	0.006	0.016	0.023	0.046
60	Participant	0.002	− 0.033	0.019	0.061
60	Back	0.010	− 0.034	0.009	0.040
17	Front	0.017	0.003	0.012	0.088
17	Participant	0.021	− 0.027	− 0.010	0.040
17	Back	0.004	− 0.011	0.000	0.018

The mean of 1-min wind velocity [m/s] in each direction and composite vector length under the indirect condition at each measurement position

### Physical measurements and PMV

**Fig. 7:**
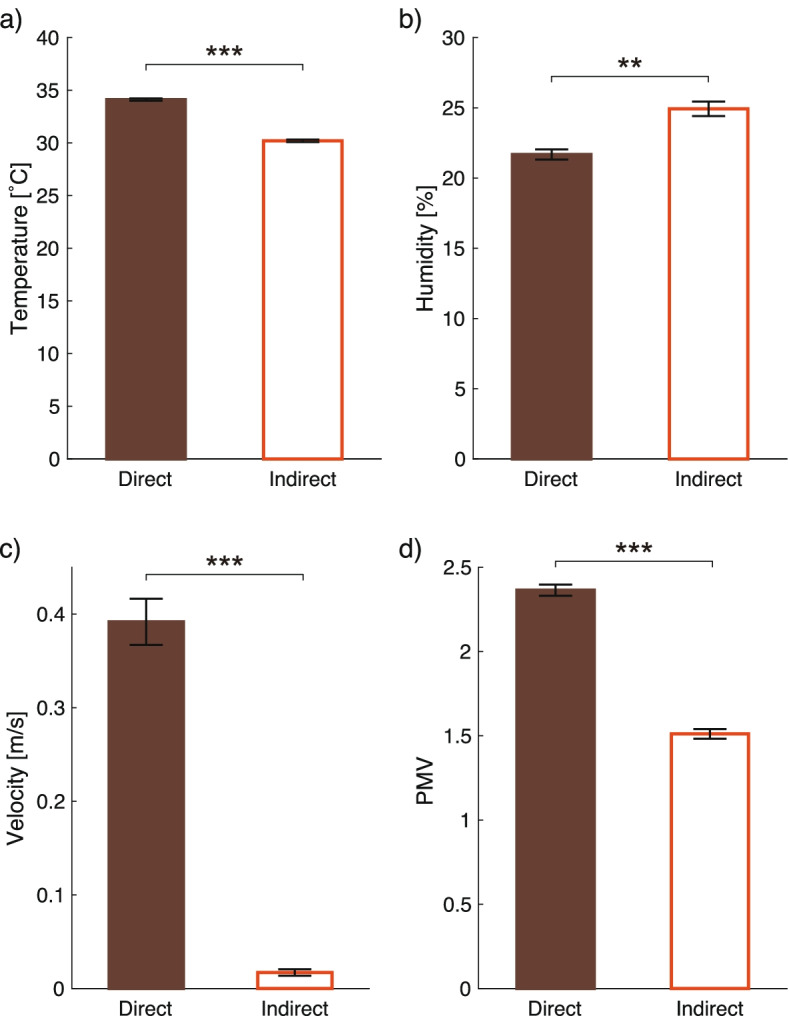
Mean and standard errors of physical data and predictive mean votes (PMV). **a** Room temperature. **b** Relative humidity. **c** Wind velocity. **d** Predictive mean votes. The combinations of an asterisk and u-shaped line show significant differences (**p < 0.01, ***p < 0.001)

Room temperature, relative humidity, and air velocities were measured at locations near the participants (Fig. 7). The mean ± SEM of the room temperature under direct and indirect airflows were 34 ± 0.19 °C and 30 ± 0.20 °C, respectively (*t*(38) = − 14, *p* < 0.001, 1−*β* > 0.99). The mean relative humidity and wind velocity under direct and indirect airflows were 22 ± 0.61% and 25 ± 0.90% (*t*(38) = 3.0, *p* = 0.0048, 1−*β* = 0.83), and 0.39 ± 0.042 m/s and 0.017 ± 0.0053 m/s (*t*(38) = − 8.9, *p* < 0.001, 1−*β* > 0.99), respectively.

To compare the thermal pleasantness and sensation in the experiment room, we compared PMV values, which predict the mean thermal comfort evaluation of a group of occupants as the combined result of environmental variables, metabolic rate, and level of clothing insulation [15]. The mean ± SEM of PMV was 2.4 ± 0.056 under direct airflow and 1.5 ± 0.043 under indirect current (*t*(37) = − 12, *p* < 0.001, 1−*β* > 0.99).

### Skin temperature

**Fig. 8:**
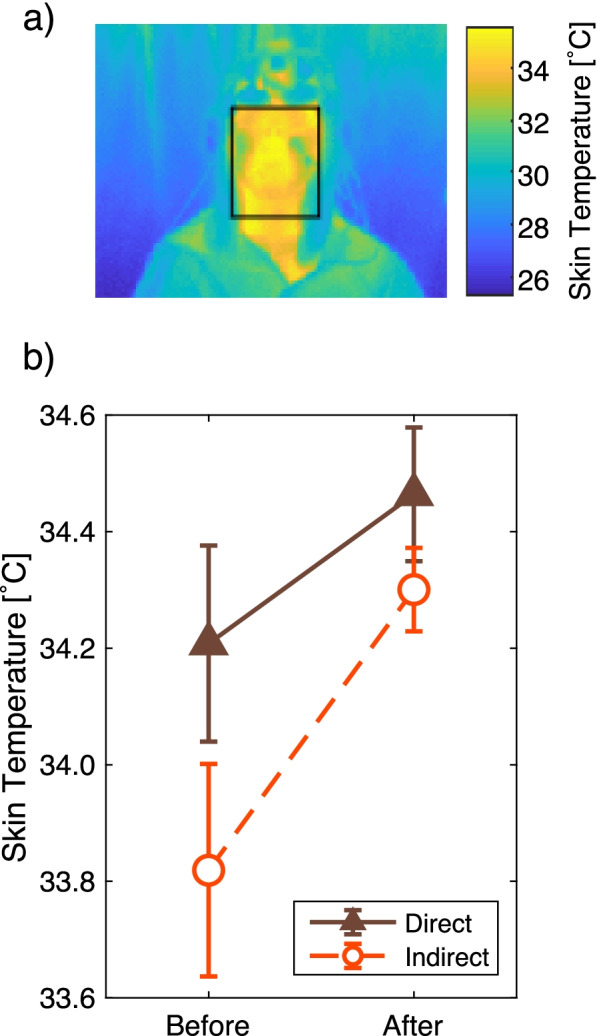
Face temperature measured by thermography images. **a** An example of face area selection. **b** Mean and standard errors of the face temperatures. The *Y*-axis shows the face temperature, which was calculated by averaging the thermography data of the face area. The *X*-axis shows the two time points, before and after the experiment

Before and after each experiment, facial skin temperature was measured with a thermographic camera. The two-way within-subject ANOVA performed with the measured face temperatures (airflow conditions × before/after condition) showed no significant effect of the airflow condition (*F*(1, 12) = 4.1, *p* = 0.065, 1−*β* = 0.52) and no interaction effect (*F*(1, 12) = 1.1, *p* = 0.32) (Fig. 8).

We confirmed the difference in skin temperatures between the sexes. The mean ± SEM skin temperature of males was 34.0 ± 0.09 °C and that of females was 34.7 ± 0.21 °C. The difference in skin temperatures among the sexes was analyzed using Welch’s two-sample *t*-test, and a significant difference was observed (*t*(4.1) = − 2.9, *p* = 0.042, 1−*β* = 0.89). The difference among the sexes did not show any interaction with the airflow conditions, which was revealed through three-way mixed ANOVA (see Supplementary Information, 3).

### EEG

In the EEG analysis, we focused on the beta and gamma frequency bands because we reported a relationship between these bands and airflow sensations in our previous study [22]. The amplitudes were analyzed for each airflow condition and frequency band.

**Fig. 9:**
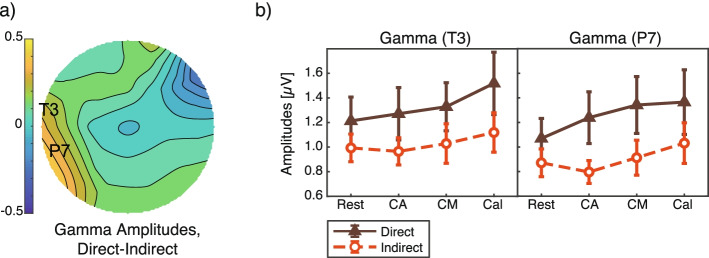
Gamma amplitudes. **a** Topography of the mean difference between direct and indirect airflow across four tasks. The electrode labels T3 and P7 indicate the analytic electrodes for which the difference between amplitudes under direct and indirect airflow was the largest. **b** Mean and standard errors of the amplitudes at T3 and P7. The *X*-axis shows the four tasks. The *Y*-axis shows the gamma amplitude, which was calculated by averaging the amplitudes across the test repetitions. The main effect of the airflow condition was significant in the two-way ANOVA (*p* < 0.05)

To show the difference of each frequency activity, the main effect of the airflow condition on each frequency band during the execution of the four tasks was analyzed by a two-way ANOVA (airflow conditions × four tasks). In terms of gamma activity at T3 and P7, the amplitudes under the indirect condition were lower than those under the direct condition (Fig. 9). These amplitudes showed a significant main effect from the airflow setting (T3: *F*(1, 132) = 4.6, *p* = 0.034, 1−*β*= 0.60; P7: *F*(1, 132) = 4.8, *p* = 0.030, 1−*β* = 0.62). There was no significant main effect from the tasks and no interaction (*F* < 0.49, *p* > 0.67).

**Fig. 10:**
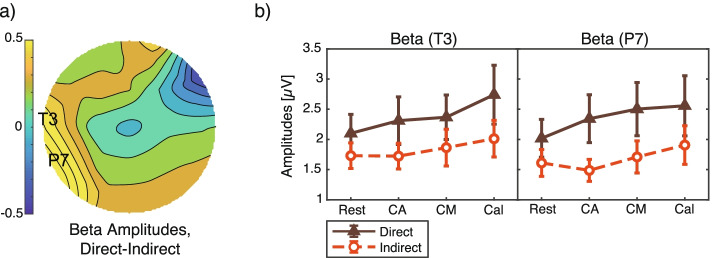
Beta amplitudes. **a** Topography of the mean difference between direct and indirect airflow across four tasks. The electrode labels T3 and P7 indicate the analytic electrodes for which the difference between amplitudes under direct and indirect airflow was the largest. **b** Mean and standard errors of the amplitudes at T3 and P7. The *X*-axis shows the four tasks. The *Y*-axis shows the gamma amplitude, which was calculated by averaging the amplitudes across the test repetitions. The two-way ANOVA showed a significant main effect from the airflow condition at P7 (*p* < 0.05) and a marginal significant effect at T3 (*p* < 0.10)

In terms of beta activity at both electrodes, the amplitudes under the indirect condition were lower than those under the direct condition (Fig. 10). The effect from the airflow condition was found to be significant on waves at P7 (*F*(1, 132) = 5.0, *p* = 0.028, 1−*β* = 0.63) but only marginal on those at T3 (*F*(1, 132) = 3.9, *p* = 0.051, 1−*β* = 0.53). Again, there was no significant main effect from the experiment tasks and no interaction in the case of beta activity (*F* < 0.43, *p* > 0.73).

### Respiration

**Fig. 11:**
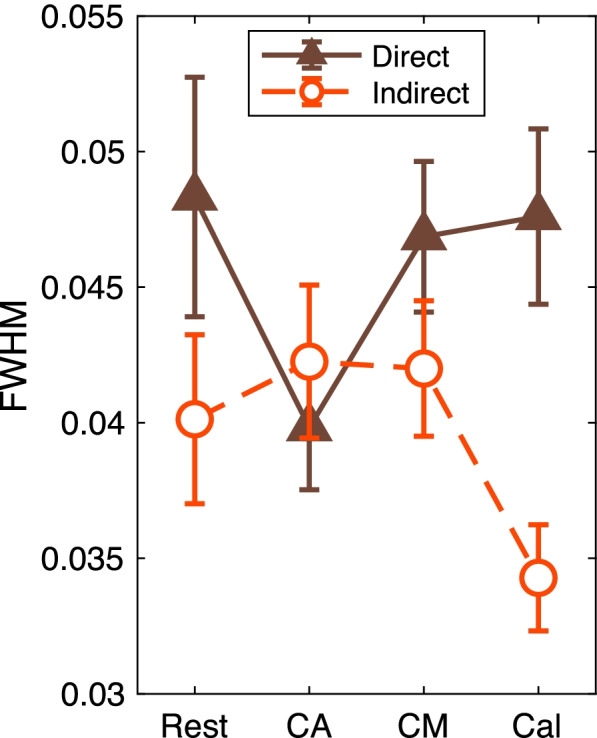
FWHM of the peak power spectral density from respiration rate. The *X*-axis shows the four tasks. The *Y*-axis shows the mean of the FWHM. The error bars are the standard errors. The two-way ANOVA showed a significant main effect from the airflow condition (*p* < 0.01)

To analyze the effects of the airflow setting on the regularity of respiration, we calculated the FWHM of the spectral density from the respiration waves. The two-way ANOVA (airflow conditions × four tasks) showed a significant main effect from the airflow condition (*F*(1, 132) = 7.4, *p* = 0.0073, 1−*β* = 0.81). Regarding the tasks, there was no significant main effect (*F*(3, 132) = 0.77, *p* = 0.51) and no interaction effect (*F*(3, 132) = 2.3, *p* = 0.084) (Fig. 11). These results indicate that the variability of respiration was lower under the indirect condition than under the direct one.

### ECG

To investigate the effects of the airflow condition on the autonomic nervous system, the ECG parameters were analyzed. HF reflects parasympathetic activity [38], and LF/HF infers sympathetic activity [39]. The two-way ANOVA showed no significant main effect or interaction effect from HF (*F* < 1.8, *p* > 0.18, 1−*β* < 0.32) or LF/HF (*F* < 0.17, *p* > 0.17, 1−*β* < 0.24).

### Subjective assessments and behavioral results

**Fig. 12:**
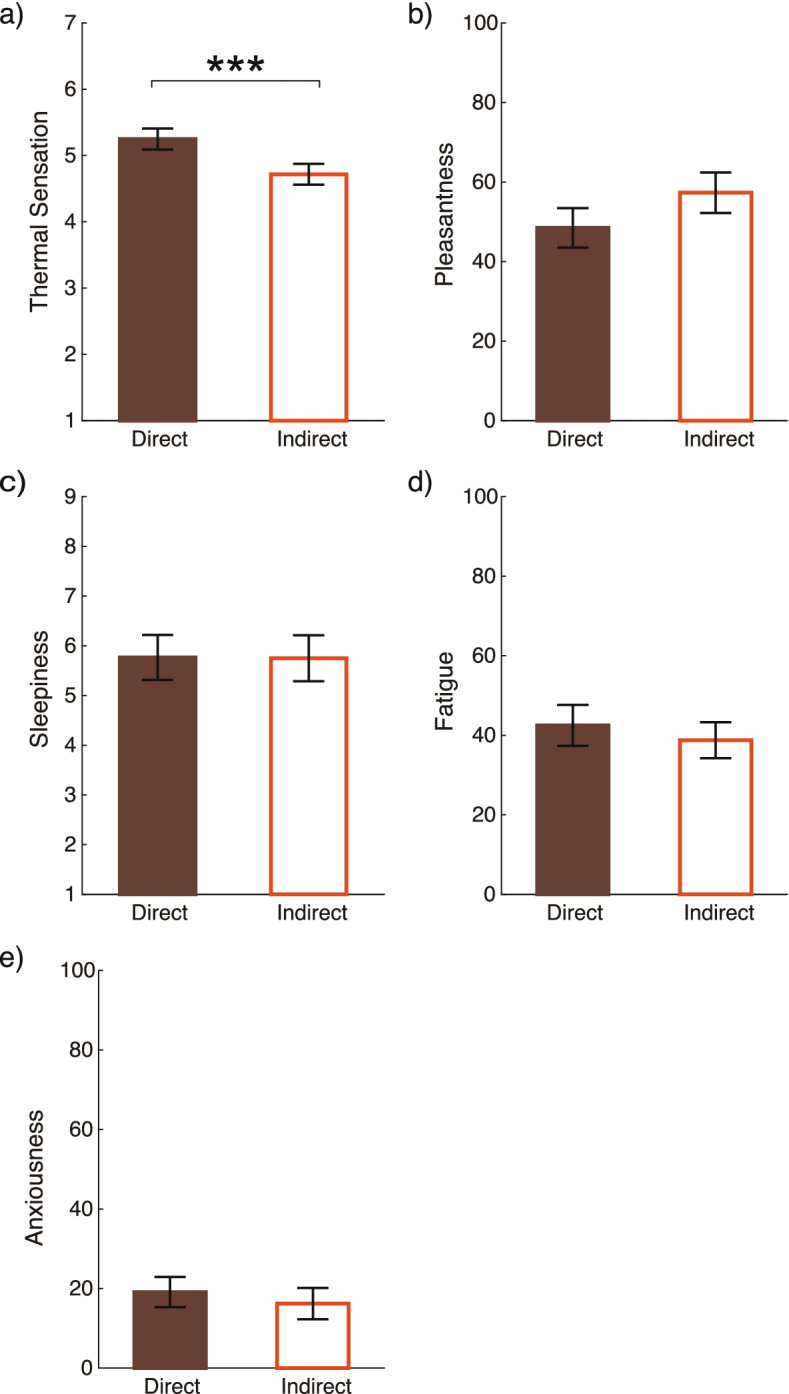
Mean and standard errors of the subjective assessments. **a** Thermal sensation. **b** Pleasantness. **c** Sleepiness. **d** Fatigue level. **e** Anxiousness. The asterisks indicate significant differences between the airflow conditions (***p < 0.001)

Under each airflow condition, the participants answered the same questionnaire, involving thermal sensation, pleasantness, fatigue, sleepiness, and anxiety. The mean scores are reported by airflow condition in Fig. 12.

The thermal sensation under indirect airflow was lower than under direct airflow, and the paired *t*-test showed a significant difference (*t*(19) = − 3.4, *p* = 0.031, 1−*β* = 0.89). The pleasantness under indirect airflow was higher than under direct airflow, but the paired *t*-test showed a marginal significance (*t*(19) = 2.0, *p* = 0.060, 1−*β* = 0.39). The other subjective assessments showed no significant difference between the two airflow conditions (*p* > 0.13).

**Fig. 13:**
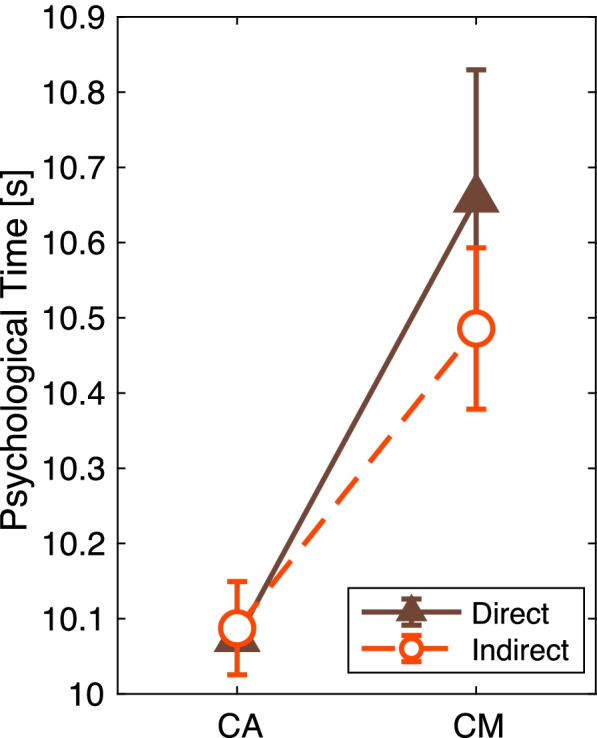
Mean and standard errors of the psychological time. The *Y*-axis indicates the mean of the psychological time, which is the time the participants perceived as 10 s. The *X*-axis indicates the two tasks. CA, counting following alerts; CM, time counting in mind

From the responses to the CA and CM tasks, based on the two-way ANOVA (airflow conditions × two tasks), the duration of psychological time showed no significant main effect or interaction effect from the airflow conditions (*p* > 0.53) (Fig. 13). This result indicates that the airflow direction did not affect psychological time.

To evaluate the mental calculation performance during the Cal assignment, the number of correct answers and the total number of mental calculations completed by each participant were counted. The total number of mental calculations was an indicator of the calculation speed. A chi-squared test did not reject the null hypothesis that the correct rate was higher under one condition than the other (*χ*^2^ = 0.13, *p* = 0.71). A paired *t*-test did not show any significant difference in the number of mental calculations performed under each condition (*t*(19) = − 0.21, *p* = 0.84). These results indicate that the airflow direction did not affect the mental calculation performance.

**Fig. 14:**
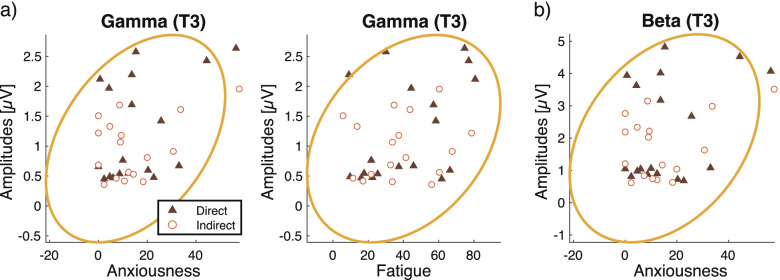
Correlation between subjective assessments and EEG amplitudes. The *X*-axes indicate subjective assessments. The *Y*-axes indicate **a** gamma and **b** beta amplitudes. Yellow lines show the 95% density ellipse. The amplitudes were averaged across the four tasks performed

The correlation between subjective assessments and EEG amplitudes was analyzed. The gamma amplitudes at T3 showed significant correlation with fatigue (*r* = 0.34, *p* = 0.048) and anxiousness (*r* = 0.41, *p* = 0.014) (Fig. 14a). The remaining subjective ratings showed no significant correlation with gamma at T3 (*|r|* < 0.29, *p* > 0.94) (Supplementary Fig. 1). The gamma amplitudes at P7 showed no significant correlation with any of the subjective assessments (*|r|* < 0.28, *p* > 0.10) (Supplementary Fig. 2).

The amplitudes of beta waves from T3 showed a significant correlation with anxiousness (*r* = 0.35, *p* = 0.037) (Fig. 14b). The other subjective ratings showed no significant correlation with beta amplitudes at T3 (*|r|* < 0.30, *p* > 0.083) (Supplementary Fig. 3). The beta waves at P7 showed no significant correlation with any of the subjective assessments (*|r|* < 0.28, *p* > 0.17) (Supplementary Fig. 4).

## Discussion

In this study, we assessed the effects of the direction of heating airflow on indoor comfort through subjective assessments and physiological measurements, including skin temperature, EEG, ECG, and respiration. The beta and gamma EEG amplitudes showed a significant main effect from the airflow condition, and respiration regularity showed the airflow effects for relaxation states. The gamma and beta amplitudes at T3 showed a significant correlation with anxiousness levels, and gamma waves also showed a meaningful correlation with fatigue. In our previous report comparing direct and indirect cooling airflows [23], we observed lower gamma and beta activities under the indirect airflow condition. The correlations suggest that the differences in physiological indices could be explained by airflow direction rather than by thermal sensation. In this study, the physiological and subjective results indicated that an indirect heating airflow induced lower mental stress than a direct air current. Some reports have concluded that people do not prefer direct airflow and high air velocity in heating [11, 12], especially in subtropical regions [9, 10]. On the other hand, other studies reported that air movement was preferred [6–8]. PMV has been widely used to estimate indoor comfort, but its equation does not factor airflow direction [15]. Therefore, we suggest that the effect of heating airflow on the mental state be evaluated through a combination of subjective and objective measurements.

We discuss the gamma activity under the different heating airflow conditions. The gamma amplitudes at T3 showed a significant correlation with anxiousness and fatigue. A possible interpretation of this correlation result is that the lower gamma amplitudes under indirect airflow reflected lower fatigue and anxiousness levels, though we could not find a significant correlation at P7. Lower gamma and beta amplitudes have also been observed under indirect cooling airflow in comparison with direct cooling airflow [23]. Thus, we can conclude that the EEG differences were caused by airflow direction, rather than by thermal sensation. We also observed the decrease in beta and gamma amplitudes in environments with no airflow, both in cooling and heating [22]. Higher gamma amplitudes reflect awareness and emotional content [40] under airflow stress [23] and have been associated with unpleasant and aversive emotions [41–43]. Furthermore, gamma amplitudes in the temporoparietal regions were found to be higher for people with an anxiety disorder than for healthy people [44]. We observed different gamma activities in the temporoparietal regions under different airflow conditions, as was also previously reported [22]. The gamma waves at the temporoparietal sites recorded in this study support such a difference in mental stress levels between direct and indirect heating airflows.

The lower beta activity observed under indirect airflow agreed with the findings of our previous study [22], and the beta amplitudes were negatively correlated with pleasantness [23]. In this study, we found a significant correlation between anxiousness ratings and beta amplitudes at T3. We can deduce that the beta waves reflected anxiousness levels of the participants, with indirect airflow inducing lower anxiousness feelings than direct airflow. Several studies have reported an association between beta activity and fatigue levels, but the sites at which differences in beta amplitudes have been recorded—right and middle frontal sites [45], left-temporal sites [46], and left and right central sites [22]—are inconsistent across these studies. In this study, we observed significant beta differences at the left temporal sites, which is consistent with a conventional report on the relationship between beta activity and mental fatigue [46]. Our results showing smaller amplitudes in the beta frequency band from left temporal areas suggest that beta activity could be a biomarker for estimating indoor comfort state.

Notably, the correlation results were insufficient to make a conclusion about the relationship between the EEG findings and mental states, although the results could provide a possible interpretation as to why the EEG differed under different airflow conditions. The *t*-test for the subjective assessments did not show significant differences, which suggests that the mental states may not differ under different airflow directions, as we discuss below.

The results of respiration regularity support our estimation that indirect heating airflow induces more relaxed states than direct airflow. The FWHM of the respiration waves was significantly smaller under indirect airflow than under direct airflow. It has been reported that mental stress could increase the variability in respiration rate, while sustained attention could decrease it [27]. A larger variability of respiration rate reflects in a larger FWHM, which was calculated from the spectral density of the respiration waves. Here, we observed a smaller FWHM under indirect airflow than under direct airflow. Thus, the analysis of respiration records confirms our prediction that indirect heating airflow can induce lower mental stress than direct airflow.

While EEG and respiratory data indicated less stress and fatigue under indirect airflow, we could not find significant differences in subjective assessments of pleasantness, sleepiness, fatigue, or anxiousness. The subjective assessment of thermal sensation under different airflow directions showed a significant difference, and it was consistent with relative PMV values. Thus, PMV could reflect thermal sensation but not mental pleasantness. This lack of correspondence between variations in most of the subjective assessments and the airflow setting may be caused by the difficulty to verbalize indoor comfort feelings, which consequently needs to be evaluated through various aspects. Otherwise, if the evaluation relied on only subjective ratings and PMV, unconscious comfort feelings would be overlooked. Related studies have reported that indoor comfort can be reflected by physiological responses, such as EEG and respiration (e.g., [24, 25, 47–49]). These physiological responses can be used as objective parameters for the evaluation of unconscious environmental comfort.

Heart rate variability showed no significant difference between airflow conditions. Other reports have found differences in heart rate variability under different environmental temperatures, such as 28 and 30 °C, 26 and 30 °C, and below [25, 49–51]. Zuo et al. reported an increase in heart rate, not heart rate variability, between 30 and 37 °C [24] and discussed that this increase may have been caused by higher heat stress. In this study, the resulting room temperature was different under the direct and indirect conditions (30 and 34 °C, respectively), but the difference was smaller than in the report by Zuo et al., and the higher temperature was not sufficient to induce heat stress. Heart rate variability may not be influenced by heating airflow direction at high room temperatures, i.e., 30+ °C. We suggest that evaluating mental state under a heating indoor environment requires several physiological measurements to be combined.

Although the environmental parameters changed depending on the airflow conditions, there was no significant difference in the participants’ face temperature. As we discussed above, we observed some EEG differences from one airflow condition to the other, but we did not observe changes in face temperature. This result suggests that EEG and respiration changes can be derived from changes in mental state but not from changes in skin temperature. We also confirmed the effects of sex on skin temperature, EEG, and respiration changes, but there was no significant interaction effect between sex and airflows (see Supplementary Information), although the mean skin temperature differed between the sexes. The mean temperature under either direct or indirect flow seemed to be different at the beginning of the experiments, though the difference decreased by the end of the tests. The order of airflow settings was pseudo-randomized across participants so that the mean temperature before the experiment could not be affected by acclimation. One possible interpretation is that the face temperature might be affected more easily by the local airflow situation before the experiment. The physical parameters were monitored but not completely controlled. The local air temperature might be different due to the airflow direction. Another possible interpretation relates to the relationship between facial temperature and mental state. It has been reported that the facial temperature decreases as a result of negative emotions [52]. According to the report, a lower facial temperature might reflect a negative reaction to direct airflow. The acceptability of air movement could increase in time but not significantly [11]. The facial temperature response to negative emotions may be moderated with time. More investigation is needed on the relationship between facial thermography and indoor comfort induced by airflow direction.

There were no significant main effects of the airflow condition on psychological time or mental calculation. Our previous investigation showed significantly different scores in the mental counting task in a heating environment under either direct airflow or no airflow [22]. In this study, we only changed airflow direction, and the difference in airflow sensation might not be sufficiently large to affect behavioral data compared to the previous settings. Furthermore, the results might be affected by a factor of task type. Zhang and de Dear reported that simple cognitive tasks are less susceptible to temperature effects than complex ones [53]. A meta-analysis and other reports support there being less effect on cognitive performance by thermal environment [14, 54]. The differences in room temperature and other physical parameters had a less severe effect on time counting and mental calculation tasks.

In our procedure, only airflow direction was modulated; however, such settings led to different room temperatures, wind directions, and relative humidity. The environmental parameters of the experiment room were monitored by the staff in a control room, but the measurement point was located nearby the indoor air conditioning unit, which was separated from the participant’s sitting point. Differences in the PMV scores resulted from these physical parameters, not only from the setting of airflow direction. Therefore, we could not conclude that the observed responses were induced only by the airflow sensation. Generally, it is very difficult to maintain the room temperature and other parameters under different airflow directions in an office or school using a commercial air conditioner. Our experimental environment was closer to general scenes. However, more restricted control will be necessary to reveal in further detail the physiological mechanism of airflow sensation.

## Conclusions

Using physiological and subjective measurements, this study investigated how the direction of airflow produced by air conditioning influences the feeling of comfort in a heating environment. The gamma and beta of EEG showed low amplitudes under indirect heating airflow, and these results indicate low emotional stress and fatigue levels. The respiration results indicate that the variability of the respiration rate was low under indirect airflow, and the results support the idea of a high relaxation state under the indirect condition. However, subjective assessments and behavioral performance could not detect the difference in mental state. These results suggest that some physiological measurements can be useful for estimating the mental state influenced by airflow direction, which cannot be determined from subjective measurements. Our procedures and results emphasize the effectiveness of physiological measurements in revealing various aspects of airflow comfort.

## Supplementary Information

**Additional file 1: Supplementary Methods.** We performed post-power analysis for the t-test and ANOVA results. To obtain the statistical power of t-tests, Cohen’s *d* values were computed as standardized effect size, using the *effsize* package in R. Each statistical power (1–β) was calculated using the corresponding Cohen’s *d*, sample size, and significance level (α = 0.05) using the *pwr* package in R. In terms of ANOVA, Cohen’s *f* values were calculated to show effect size of the factors. The post-hoc power was calculated using effect size, sample size, and significant level (alpha = 0.05) using the pwr2 package in R. **Supplementary Table 1.** Cohen’s *d* values for effect size and powers obtained by post-power analysis for t-test. **Supplementary Table 2.** Cohen’s *d* values for effect size and powers obtained by post-power analysis for subjective assessments. **Supplementary Table 3.** Cohen’s *f* values for effect size and powers for the main effects obtained by post-power analysis for two-way ANOVA of EEG. **Supplementary Table 4.** Cohen’s *f* values for effect size and powers for the main effects obtained by post-power analysis for two-way ANOVA of thermography. **Supplementary Table 5.** Cohen’s *f* values for effect size and powers for the main effects obtained by post-power analysis for two-way ANOVA of respiration. **Supplementary Table 6.** Cohen’s *f* values for effect size and powers for the main effects obtained by post-power analysis for two-way ANOVA of ECG parameters, HF and LF/HF. **Supplementary Figure 1.** Scatter plots of gamma amplitudes at T3 as a function of each subjective assessment. Yellow lines show the 95% density ellipse. The amplitudes were averaged across the four tasks performed. There was no significant correlation (*P* > 0.05). **Supplementary Figure 2.** Scatter plots of gamma amplitudes at P7 as a function of each subjective assessment. Yellow lines show the 95% density ellipse. The amplitudes were averaged across the four tasks performed. There was no significant correlation (*P* > 0.05). **Supplementary Figure 3.** Scatter plots of beta amplitudes at T3 as a function of each subjective assessment. Yellow lines show the 95% density ellipse. The amplitudes were averaged across the four tasks performed. There was no significant correlation (*P* > 0.05). **Supplementary Figure 4.** Scatter plots of beta amplitudes at P7 as a function of each subjective assessment. Yellow lines show the 95% density ellipse. The amplitudes were averaged across the four tasks performed. There was no significant correlation (*P* > 0.05).

## Data Availability

The datasets generated and/or analyzed during the current study are not publicly available due to a privacy policy but are available from the corresponding author on reasonable request.

## Abbreviations

ANOVA: Analysis of variance
Cal: Mental calculation
CA: Time counting following alerts
CM: Time counting in mind
DFT: Discrete Fourier transform
ECG: Electrocardiography
EEG: Electroencephalography
EOG: Electrooculogram
FWHM: Full width at half maximum
fMRI: Functional magnetic resonance imaging
PMV: Predicted mean vote
SpO_2_: Peripheral oxygen saturation
HF: High frequency
LF: Low frequency
