# Supplementary material for: Physiological comfort evaluation under different airflow directions in a heating environment
Source: J Physiol Anthropol. 2022 Apr 15;41:16. doi: 10.1186/s40101-022-00289-x (PMC9012013; doi:10.1186/s40101-022-00289-x)
Supplement: Supplementary file 1 — Additional file 1: Supplementary Methods. We performed post-power analysis for the t-test and ANOVA results. To obtain the statistical power of t-tests, Cohen’s d values were computed as standardized effect size, using the effsize package in R. Each statistical power (1–β) was calculated using the corresponding Cohen’s d, sample size, and significance level (α = 0.05) using the pwr package in R. In terms of ANOVA, Cohen’s f values were calculated to show effect size of the factors. The post-hoc power was calculated using effect size, sample size, and significant level (alpha = 0.05) using the pwr2 package in R. Supplementary Table 1. Cohen’s d values for effect size and powers obtained by post-power analysis for t-test. Supplementary Table 2. Cohen’s d values for effect size and powers obtained by post-power analysis for subjective assessments. Supplementary Table 3. Cohen’s f values for effect size and powers for the main effects obtained by post-power analysis for two-way ANOVA of EEG. Supplementary Table 4. Cohen’s f values for effect size and powers for the main effects obtained by post-power analysis for two-way ANOVA of thermography. Supplementary Table 5. Cohen’s f values for effect size and powers for the main effects obtained by post-power analysis for two-way ANOVA of respiration. Supplementary Table 6. Cohen’s f values for effect size and powers for the main effects obtained by post-power analysis for two-way ANOVA of ECG parameters, HF and LF/HF. Supplementary Figure 1. Scatter plots of gamma amplitudes at T3 as a function of each subjective assessment. Yellow lines show the 95% density ellipse. The amplitudes were averaged across the four tasks performed. There was no significant correlation (P > 0.05). Supplementary Figure 2. Scatter plots of gamma amplitudes at P7 as a function of each subjective assessment. Yellow lines show the 95% density ellipse. The amplitudes were averaged across the four tasks performed. There was no significant correlation (P [file 40101_2022_289_MOESM1_ESM.docx]

Supplementary Material

Physiological comfort evaluation under different airflow directions in a heating environment

Kaori Tamura^1,2^, Sayaka Matsumoto^1^, YuHsuan Tseng^3^, Takayuki Kobayashi^4^, Jun’ichi Miwa^5^, Ken’ichi Miyazawa^4^, Soichiro Matsumoto^4^, Seiji Hiramatsu^4^, Hiroyuki Otake ^4^, and Tsuyoshi Okamoto^1,3*^

*Corresponding author: okamoto@artsci.kyushu-u.ac.jp

^1^Faculty of Arts and Science, Kyushu University, 744 Motooka, Nishi-ku, Fukuoka, 819-0395, Japan

^2^Department of Information and Systems Engineering, Faculty of Information Engineering, Fukuoka Institute of Technology, 3-30-1 Wajiro-higashi, Higashi-ku, Fukuoka, 811-0295, Japan

^3^Graduate School of Systems Life Sciences, Kyushu University, 744 Motooka, Nishi-ku, Fukuoka, 819-0395, Japan

^4^Mitsubishi Heavy Industries Thermal Systems LTD., 3-1, Asahi, Nishi-biwajima-cho, Kiyosu, Aichi, 452-8561, Japan

^5^Research and Innovation Center, Mitsubishi Heavy Industries LTD., 1, Aza Kanda, Iwatsuka-cho, Nakamura-ku, Nagoya, Aichi, 453-8515, Japan

# Supplementary Methods

We performed post-power analysis for the t-test and ANOVA results. To obtain the statistical power of t-tests, Cohen’s *d* values were computed as standardized effect size, using the *effsize* package in R. Each statistical power (1–β) was calculated using the corresponding Cohen’s *d,* sample size, and significance level (α = 0.05) using the *pwr* package in R. In terms of ANOVA, Cohen’s *f* values were calculated to show effect size of the factors. The post-hoc power was calculated using effect size, sample size, and significant level (alpha = 0.05) using the *pwr2* package in R.

# Supplementary Tables and Figures

|  | Cohen’s *d* | Power (1–β) |
| --- | --- | --- |
| room temperature | 4.50 | >0.99 |
| relative humidity | 0.95 | 0.83 |
| air velocity | 2.82 | >0.99 |
| PMV | 3.89 | >0.99 |

**Supplementary Table 1**. Cohen’s *d* values for effect size and powers obtained by post-power analysis for t-test.

|  | Cohen’s *d* | Power (1–β) |
| --- | --- | --- |
| Thermal Sensation | 0.76 | 0.89 |
| Pleasantness | 0.39 | 0.39 |
| Sleepiness | 0.17 | 0.11 |
| Fatigue | -0.0082 | 0.05 |
| Anxiousness | -0.17 | 0.11 |

**Supplementary Table 2**. Cohen’s *d* values for effect size and powers obtained by post-power analysis for subjective assessments.

|  | Airflow | | Tasks | |
| --- | --- | --- | --- | --- |
|  | Cohen’s *f* | Power (1–β) | Cohen’s *f* | Power (1–β) |
| Gamma T3 | 0.19 | 0.60 | 0.084 | 0.12 |
| Gamma P7 | 0.19 | 0.62 | 0.11 | 0.16 |
| Beta T3 | 0.17 | 0.53 | 0.096 | 0.14 |
| Beta P7 | 0.19 | 0.63 | 0.099 | 0.14 |

**Supplementary Table 3.** Cohen’s *f* values for effect size and powers for the main effects obtained by post-power analysis for two-way ANOVA of EEG.

|  | Cohen’s *f* | Power (1–β) |
| --- | --- | --- |
| Airflow | 0.28 | 0.52 |
| Before/After | 0.56 | 0.98 |

**Supplementary Table 4.** Cohen’s *f* values for effect size and powers for the main effects obtained by post-power analysis for two-way ANOVA of thermography.

|  | Cohen’s *f* | Power (1–β) |
| --- | --- | --- |
| Airflow | 0.24 | 0.81 |
| Tasks | 0.13 | 0.23 |

**Supplementary Table 5**. Cohen’s *f* values for effect size and powers for the main effects obtained by post-power analysis for two-way ANOVA of respiration.

|  | Airflow | | Tasks | |
| --- | --- | --- | --- | --- |
|  | Cohen’s *f* | Power (1–β) | Cohen’s *f* | Power (1–β) |
| HF | 0.12 | 0.29 | 0.16 | 0.32 |
| LF/HF | 0.10 | 0.24 | 0.11 | 0.18 |

**Supplementary Table 6.** Cohen’s *f* values for effect size and powers for the main effects obtained by post-power analysis for two-way ANOVA of ECG parameters, HF and LF/HF.


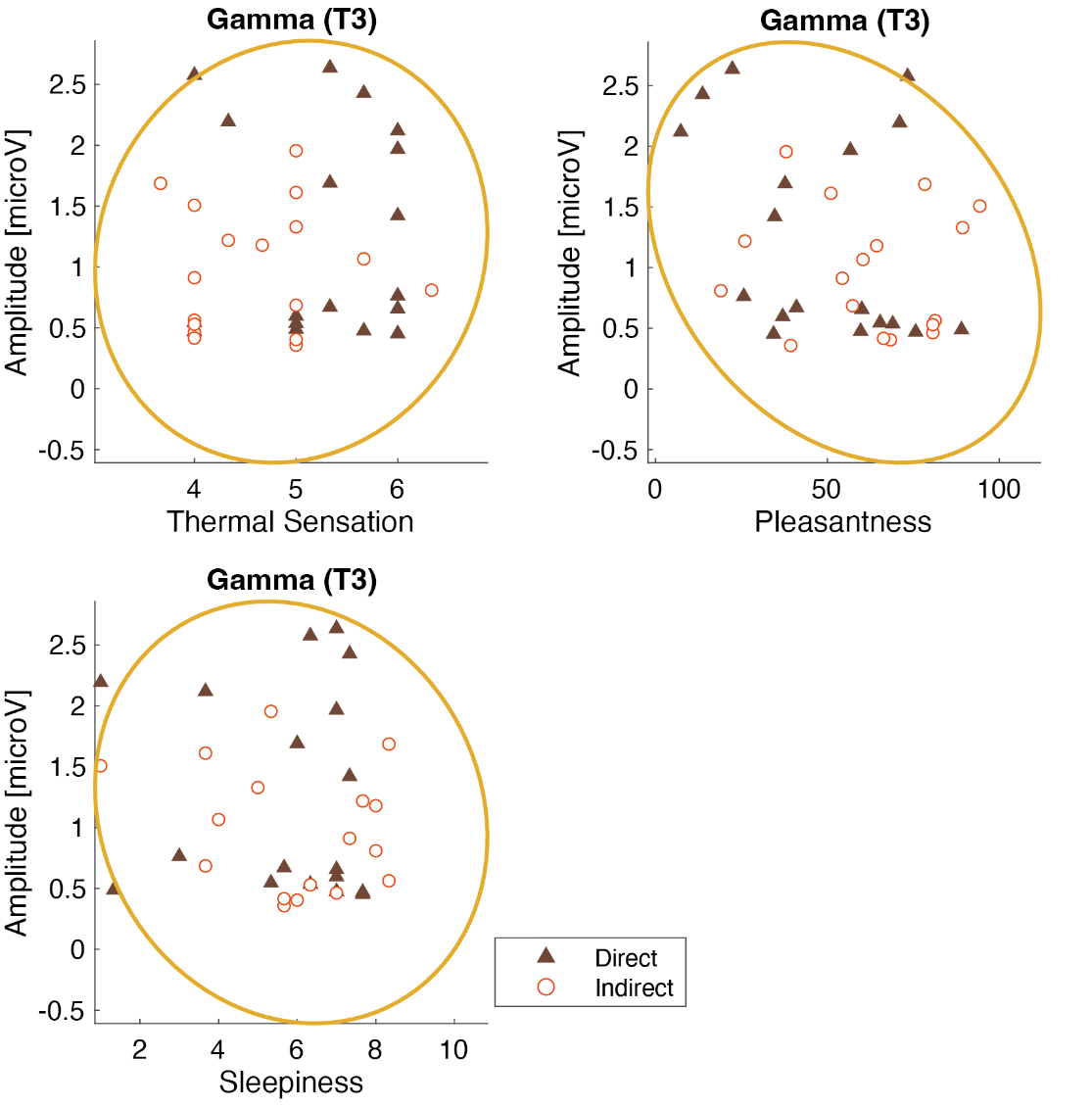


**Supplementary Figure 1.** Scatter plots of gamma amplitudes at T3 as a function of each subjective assessment. Yellow lines show the 95% density ellipse. The amplitudes were averaged across the four tasks performed. There was no significant correlation (*P* > 0.05).


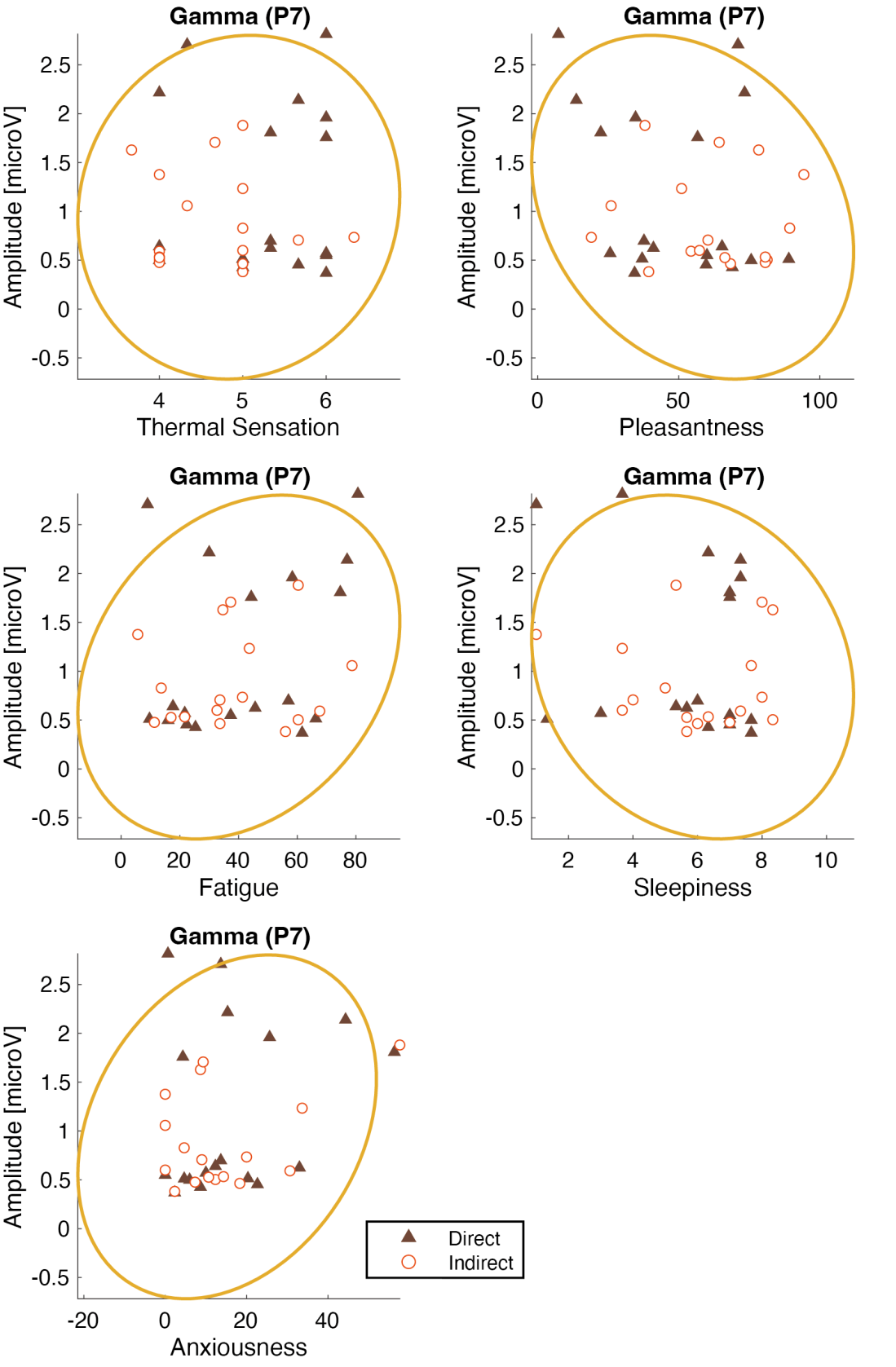


**Supplementary Figure 2.** Scatter plots of gamma amplitudes at P7 as a function of each subjective assessment. Yellow lines show the 95% density ellipse. The amplitudes were averaged across the four tasks performed. There was no significant correlation (*P* > 0.05).


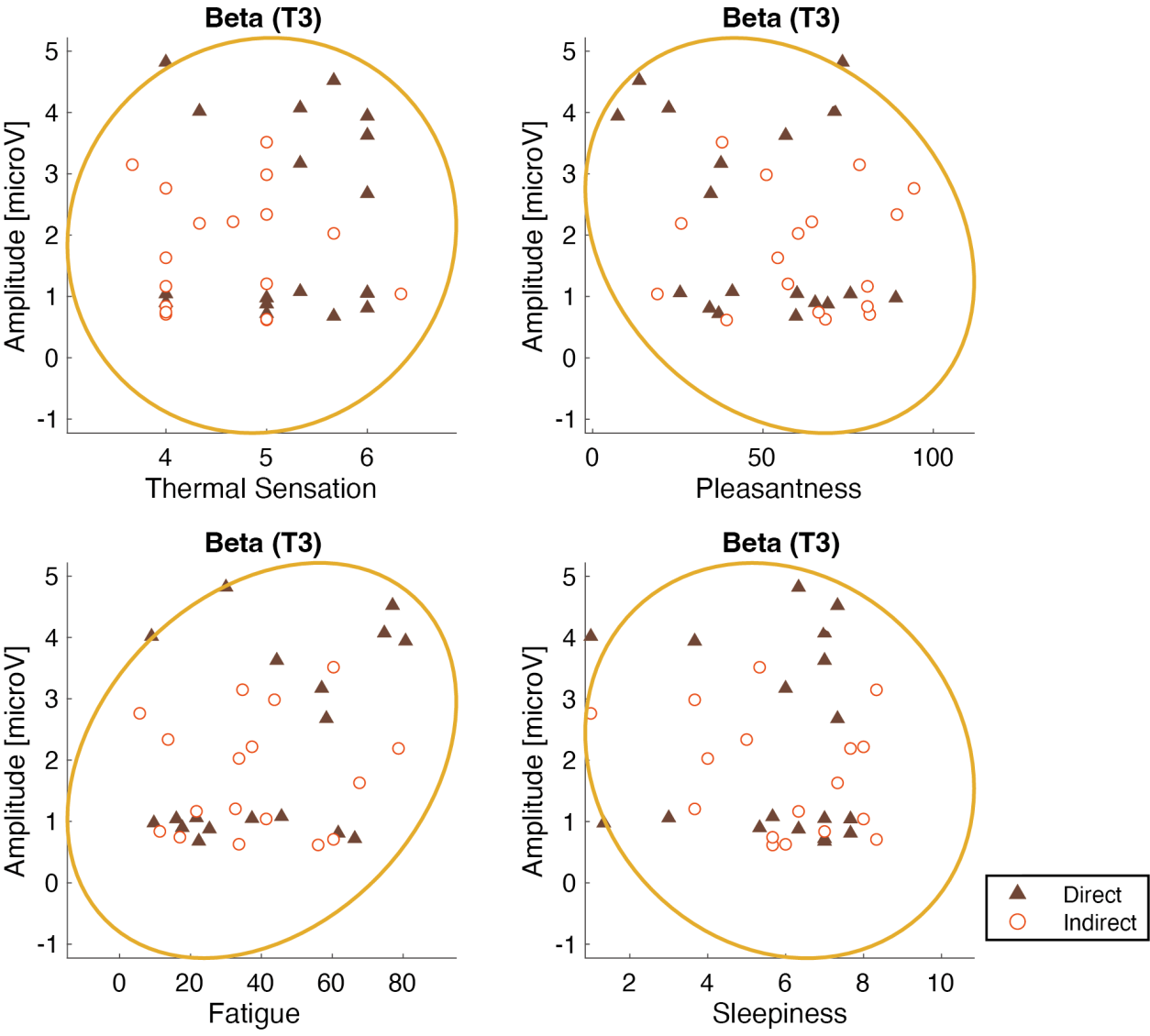


**Supplementary Figure 3.** Scatter plots of beta amplitudes at T3 as a function of each subjective assessment. Yellow lines show the 95% density ellipse. The amplitudes were averaged across the four tasks performed. There was no significant correlation (*P* > 0.05).


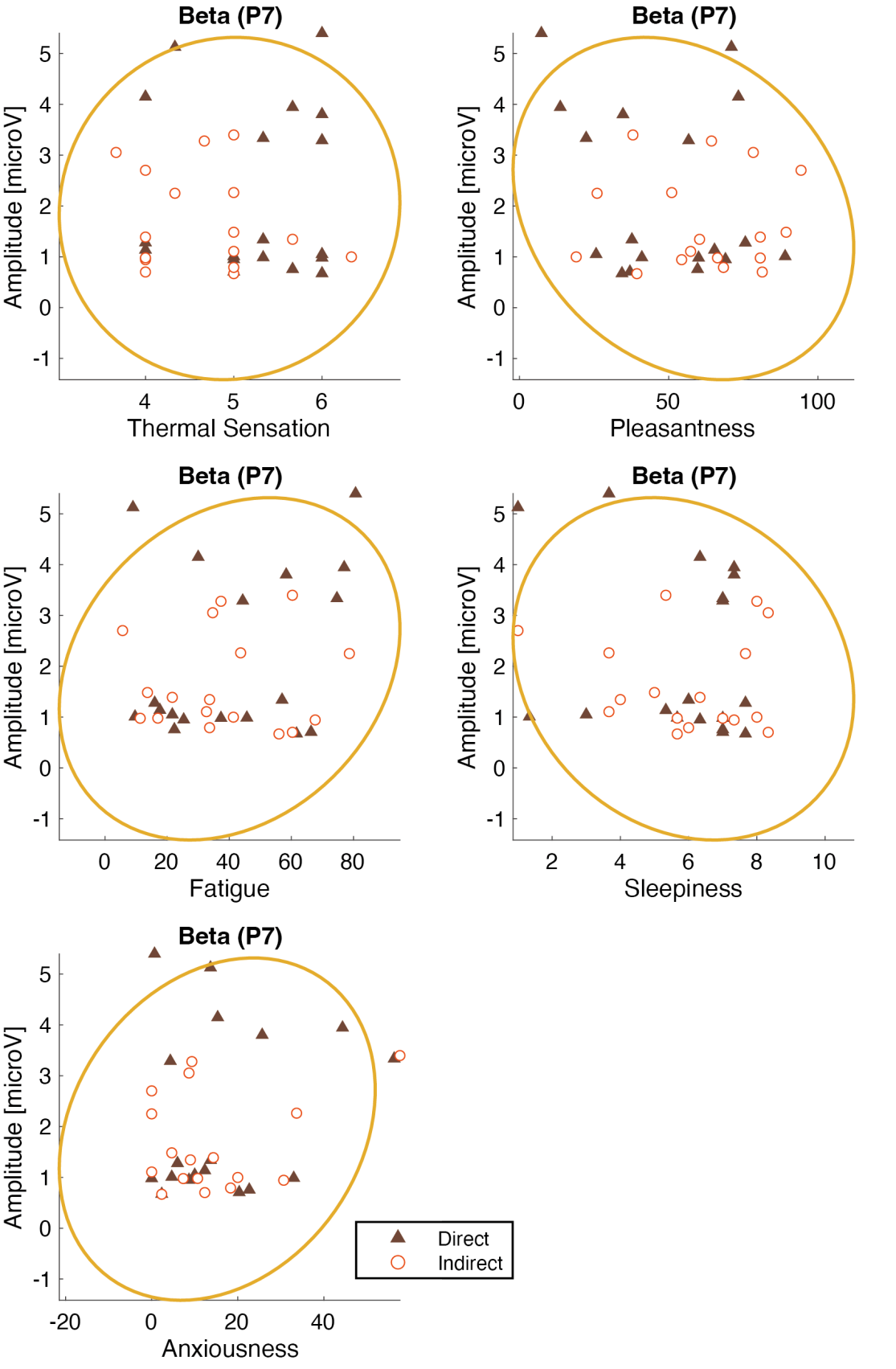


**Supplementary Figure 4.** Scatter plots of beta amplitudes at P7 as a function of each subjective assessment. Yellow lines show the 95% density ellipse. The amplitudes were averaged across the four tasks performed. There was no significant correlation (*P* > 0.05).

# Supplementary data

# Effect of sex on skin temperature, EEG, and subjective assessments

To confirm the difference in skin temperatures among the sexes, we also performed another ANOVA including the sex effect. The three-way mixed ANOVA (airflow conditions × before/after condition × sex) showed a significant main effect of sex (F(1,11) = 12.4, *p* = 0.048), but no significant main effect of airflow (F(1, 11) = 2.8, *p* = 0.12) and no interaction between sex and airflow condition was observed (F(1,11) = 0.11, *p* = 0.75). There was no significant effect of airflow conditions between men and women, although the skin temperature showed a difference.

In terms of EEG, the effect of sex difference was analyzed by a three-way mixed ANOVA (airflow conditions × before/after condition × sex) at each frequency band and for each electrode. The gamma and beta activities did not show any significant main effect of sex (F(1,17) < 0.67, p > 0.42), and no interaction effect between sex and airflow conditions was observed (F(1,17) < 0.044, p > 0.83).

The sex effect of subjective assessments was analyzed by two-way mixed ANOVA (airflow conditions × sex). No significant main effect of sex (F(1,18) < 2.1, *p* > 0.16) and no interaction (F(1,18) < 0.42. *p* > 0.52) in each questionnaire was observed.
